# Effect of hyperoside on cervical cancer cells and transcriptome analysis of differentially expressed genes

**DOI:** 10.1186/s12935-019-0953-4

**Published:** 2019-09-09

**Authors:** Weikang Guo, Hui Yu, Lu Zhang, Xiuwei Chen, Yunduo Liu, Yaoxian Wang, Yunyan Zhang

**Affiliations:** 1 Department of Gynecology, Harbin Medical University Cancer Hospital, No. 150 Haping Road, Nangang District, Harbin, 150081 Heilongjiang Province China; 2 Department of Cardiopulmonary Function, Harbin Medical University Cancer Hospital, Harbin, 150081 Heilongjiang Province China

**Keywords:** Hyperoside, Cervical cancer, RNA-Seq, Differentially expressed genes, Gene ontology, Kyoto Encyclopedia of Genes and Genomes, Protein–protein interactions network, Survival analysis

## Abstract

**Background:**

Hyperoside (Hy) is a plant-derived quercetin 3-d-galactoside that exhibits inhibitory activities on various tumor types. The objective of the current study was to explore Hy effects on cervical cancer cell proliferation, and to perform a transcriptome analysis of differentially expressed genes.

**Methods:**

Cervical cancer HeLa and C-33A cells were cultured and the effect of Hy treatment was determined using the Cell Counting Kit-8 (CCK-8) assay. After calculating the IC50 of Hy in HeLa and C-33A cells, the more sensitive to Hy treatment cell type was selected for RNA-Seq. Differentially expressed genes (DEGs) were identified by comparing gene expression between the Hy and control groups. Candidate genes were determined through DEG analysis, protein interaction network (PPI) construction, PPI module analysis, transcription factor (TF) prediction, TF-target network construction, and survival analysis. Finally, the key candidate genes were verified by RT-qPCR and western blot.

**Results:**

Hy inhibited HeLa and C33A cell proliferation in a dose- and time-dependent manner, as determined by the CCK-8 assay. Treatment of C-33A cells with 2 mM Hy was selected for the subsequent experiments. Compared with the control group, 754 upregulated and 509 downregulated genes were identified after RNA-Seq. After functional enrichment, 74 gene ontology biological processes and 43 Kyoto Encyclopedia of Genes and Genomes pathways were obtained. According to the protein interaction network (PPI), PPI module analysis, TF-target network construction, and survival analysis, the key genes *MYC*, *CNKN1A*, *PAX2*, *TFRC*, *ACOX2*, *UNC5B*, *APBA1*, *PRKACA*, *PEAR1*, *COL12A1*, *CACNA1G*, *PEAR1*, and *CCNA2* were detected. RT-qPCR was performed on the key genes, and Western blot was used to verify *C*-*MYC* and *TFRC*. *C*-*MYC* and *TFRC* expressions were lower and higher than the corresponding values in the control group, respectively, in accordance with the results from the RNA-Seq analysis.

**Conclusion:**

Hy inhibited HeLa and C-33A cell proliferation through *C*-*MYC* gene expression reduction in C-33A cells and *TFRC* regulation. The results of the current study provide a theoretical basis for Hy treatment of cervical cancer.

## Background

Cervical cancer is a malignant epithelial tumor that occurs in the cervix. Most cervical cancers can be screened early by cervical cytology and virology. Moreover, human papillomavirus (Hpv) vaccination has emerged as an effective method for cervical cancer prevention [1]. However, due to inadequate screening programs in many parts of the world, cervical cancer remains one of the most common cancer types in females [2, 3]. Surgery is the main method for early treatment. Radiotherapy and chemotherapy are further therapy options. Women with cervical, especially advanced or recurrent, cancer are commonly treated using chemotherapy [4]. Recently, several reports have implicated traditional Chinese medicines in the treatment of cervical cancer. For example, ferulic acid inhibits the proliferation, invasion, and autophagy of cervical cancer cells, and induces cell cycle arrest [5]. Moreover, casticin induces G0/G1 cell cycle arrest and apoptosis in gallbladder cancer [6]. Hyperoside (Hy) is a flavonoid found mainly in Chinese herbal medicines. It exhibits anti-inflammatory, anti-oxidative, and vascular protective effects. Several recent studies demonstrated an anticancer effect of Hy in a variety of tumor types. Thus, Hy increased apoptosis and autophagy in pancreatic cancer cells [7]. Another study described Hy-mediated inhibition of human osteosarcoma cell proliferation and promotion of osteogenic differentiation [8]. Yet another study implicated Hy in the caspase-3, p53, and nuclear factor-kappa B (NF-κB) signaling pathways, which induce apoptosis and inhibit lung cancer cell proliferation [9, 10]. In gynecological oncology, Hy induces endometrial cancer cell apoptosis through the mitochondrial pathway [11]. However, Hy effect on cervical cancer development and the molecular mechanism implicated are unclear.

In the current study, the effect of Hy on two cervical cancer cell lines was determined using cytological methods, to detect changes in the cell proliferation index. Differentially expressed genes (DEGs) were identified by RNA sequencing (RNA-seq), comparing untreated and Hy-treated cells. Further analyses of the DEGs were conducted to explore the specific mechanism of Hy action on cervical cancer cells.

## Methods

### Cell culture

HeLa and C-33A cells (both acquired from the Chinese Academy of Sciences Shanghai Cell Bank) were cultured in Dulbecco’s modified Eagle’s medium (Gibco,Waltham, MA, USA) supplemented with 10% fetal bovine serum. They were inoculated in 96-well plates, cultured at 37 °C for 24 h, and then divided into seven groups. One group was untreated, whereas the remaining groups were treated with 0.25, 0.5, 1, 2, 4, or 8 mM Hy (Solarbio, Beijing, China) for 24, 48, or 72 h.

### Cell viability and IC50 determination

Cell viability was determined using the Cell Counting Kit-8 (CCK-8) assay at 24, 48, and 72 h. At each time point, 100 µL CCK-8 (Beyotime Bio, Shanghai, China) was added to each well of a 96-well plate, which was then placed in a 37 °C, 5% CO_2_ incubator. HeLa and C-33A cells were incubated for 0.5 and 2 h, respectively, in the dark. The absorbance of each well was measured at 450 nm using an EPOCH microplate reader (Gene Company Limited). The half-inhibitory concentration (IC50) was calculated with GraphPad (version 5.0), and the cell line exhibiting higher sensitivity to Hy treatment was selected for follow-up experiments.

### RNA-Seq and data preprocessing

Cells were divided into two groups for this experiment: a Hy-treated and a blank control group; the experiment was repeated three times with independent biological samples. Total RNA was extracted using TRIzol (TaKaRa Bio, Dalian, China), and the extracted RNA was sent to Shanghai New Bioinformatics Co., Ltd. to construct a cDNA library using an Illumina HiSeqTM 2000 platform for double-end PE150 sequencing with 6G data per sample. Unreliable bases and reads were filtered out to obtain clean data for the six samples. The TopHat software (version 2.1.0) was used to locate clean reads on the human reference genome (GENCODE download, GRCh38) [12]. The read count information on each gene alignment was obtained using the htseq-count tool (version 0.9.1) based on the human gene annotation information provided by GENCODE (Release 25).

### Inter-sample expression level and principal component analysis

The cor function of the R software (version: 3.4.1) was used to calculate the similarity between the two samples in each experiment. The prcomp function of the R software was utilized to reduce the dimensionality of the data. The ggfortify package (version: 0.4.6) created PCA plots for principal component analysis.

### DEG screening

First, the raw count was normalized using the TMM algorithm in the edgeR package [13, 14] (version: 3.4). Second, the mean–variance relationship was modeled with the exact weighting method (voom) provided by the limma package [15] (version: 3.36.2). Then, using linear regression and empirical Bayesian methods provided by the limma package, differential expression analysis was performed on the Hy and control groups. The differential expression threshold for DEGs was set to P < 0.05, |logFC| > 0.585.

### Kyoto Encyclopedia of Genes and Genomes (KEGG) and Gene Ontology (GO)

GO [16] functional annotation and KEGG [17] enrichment analysis of the DEGs were performed using the DAVID (version 6.8, https://david-d.ncifcrf.gov/) [18]. P < 0.05 and enrichment count of at least 3 were considered thresholds for significant enrichment results.

### Protein–protein interaction network (PPI) and PPI module analysis

The STRING (version 10.0, http://www.string-db.org/) database [19] was used to predict whether gene-encoded proteins interact with each other. A PPI network was constructed for the DEGs with the STRING database (parameter setting: species = homo; PPI score = 0.9). After obtaining the PPI relationship, a network diagram was constructed with Cytoscape (version 3.4.0, http://chianti.ucsd.edu/cytoscape-3.4.0/) [20]. CytoNCA plugin [21] (version 2.1.6, parameter setting: default) for Cytoscape was used to analyze the topological properties of the node network. The hub protein in the PPI network was obtained by ranking the network topology properties for each node.

The MCODE plugin [22] (version 1.5.1, parameter setting: default) for Cytoscape was used to screen protein complexes or functional modules. The modules with a score > 5 in the screening result were analyzed for KEGG path enrichment using the R package clusterProfiler [23] (version: 3.8.1).

### Transcription factor prediction

The genes corresponding to the proteins identified in the PPI network were used as candidate genes, and transcription factors (TFs) were predicted with the TRRUST (version 2, http://www.grnpedia.org/trrust/, threshold setting: q-value < 0.05, number of target genes ≥ 2) [24]. The predicted TFs were compared with the DEGs to obtain differential TFs, and the transcription regulatory network (TF-target network) was constructed utilizing the Cytoscape software.

### Survival analysis of key genes

The dataset for survival analysis was obtained from the UCSC database (http://xena.ucsc.edu/) [25], which contains TCGA-related data. Cancer samples with available patient survival information (n = 291) were selected, and the TCGA cervical cancer clinical data were used to extract the clinical information related to prognosis. The genes corresponding to the hub proteins obtained from the PPI network and the TFs in the TF-target regulatory network were utilized as candidate genes, and candidate gene expression values were screened from the TCGA. The median values were divided into two groups (high expression and low expression). A log-rank statistical test was performed, and the threshold *P* value was set to < 0.05. The relationship between candidate genes and patient prognosis was analyzed, and a Kaplan–Meier survival curve was plotted.

### RT-qPCR analysis

Key genes for RT-qPCR verification were selected based on the PPI networks, topological properties, TF analyses, logFC, and degree ranking data. RNA extraction was performed using Trizol (TaKaRa Bio, Dalian, China), and cDNA was synthesized with PrimeScript RT Master Mix (TaKaRa Bio, Tokyo, Japan). Subsequently, amplification was carried out based on the Power SYBR Green PCR Master Mix (Thermo Fisher Scientific, Waltham, MA, USA). After an initial denaturation step of 10 min at 95 °C, the product was routinely examined using a dissociation curve, and the amount of transcript was compared with the relative Ct method with glyceraldehyde 3-phosphate dehydrogenase (GAPDH) as an internal reference control. The 2^−ΔΔ Cq^ method was utilized for analysis of the experimental data. Primers and primer sequences for each gene are provided in Table 1.

**Table 1:** Primers and primer sequences for each gene analyzed with RT-qPCR

Genes	Primer sequences (5′-3′)
APBA1-hF	TTATTCCCAGGCTTGGCACC
APBA1-hR	TCGGAACGGCTAGGAGAGAA
CCNA2-hF	CGCTGGCGGTACTGAAGTC
CCNA2-hR	GAGGAACGGTGACATGCTCAT
CDKN1A-hF	CGATGGAACTTCGACTTTGTCA
CDKN1A-hR	GCACAAGGGTACAAGACAGTG
COL12A1-hF	CAAAGGAGGCAATACTCTCACAG
COL12A1-hR	GAAGGTG`CTTCAACATCGTCT
MYC-h F	CCTGGTGCTCCATGAGGAGAC
MYC-h R	CAGACTCTGACCTTTTGCCAGG
PAX2-hF	TCAAGTCGAGTCTATCTGCATCC
PAX2-hR	CATGTCACGACCAGTCACAAC
PEAR1-hF	TACCGGACCGTGTACCGTC
PEAR1-hR	CACACTCACTGGAACAGTCGT
RB1-hF	CTCTCGTCAGGCTTGAGTTTG
RB1-hR	GACATCTCATCTAGGTCAACTGC
TFRC-hF	ACCATTGTCATATACCCGGTTCA
TFRC-hR	CAATAGCCCAAGTAGCCAATCAT
PRKACA-hF	ACCCTGAATGAAAAGCGCATC
PRKACA-hR	CGTAGGTGTGAGAACATCTCCC
ACOX2-hF	CGCCTGGGTTGGTTAGAAGAT
ACOX2-hR	CTGAGGGCTCTCACGAAGAC
CACNA1G-hF	ACACTTGGAACCGGCTTGAC
CACNA1G-hR	AGCACACGGACTGTCCTGA
UNC5B-hF	GTCGGACACTGCCAACTATAC
UNC5B-hR	CCGCCATTCACGTAGACGAT
GAPDH-hF	TGACAACTTTGGTATCGTGGAAGG
GAPDH-hR	AGGCAGGGATGATGTTCTGGAGAG

### Western blot analysis4

The *MYC* and *TFRC* genes, which were identified by RT-qPCR, were selected for western blot analysis. Hy-treated cells were lysed with RIPA9 (Beyotime Bio, Shanghai, China), and the bicinchoninic acid (BCA; Thermo Fisher Scientific) reaction was performed to quantify protein concentrations. Equal protein amounts were resolved using 10% SDS-PAGE and transferred to polyvinylidene fluoride membranes (Millipore, Billerica, MA, USA). The membranes were blocked with 5% skim milk for 1 h, and then one of the following primary antibodies was added: anti-c-Myc rabbit monoclonal antibody (mAb; 57 kDa, 1:1000 dilution, Abcam, Cambridge, MA, USA,), anti-transferrin receptor (TFRC) rabbit monoclonal antibody (45 kDa, 1:5000 dilution, Abcam, Cambridge, MA, USA), or anti-GAPDH murine monoclonal antibody (36 kDa, 1:1000 dilution, Santa Cruz Biotechnology, CA, USA). After an overnight incubation at 4 °C, a secondary antibody (rabbit mAb, 1:10,000 or murine mAb, 1:5000) was added and incubated for 2 h at 37 °C. After development with the Millipore ECL system, the optical density of the target strips was analyzed using a chemiluminescent system (Tanon, Shanghai, China).

### Statistical analysis

All experiments were replicated at least 3 times, and the data are presented as mean ± standard deviation. The results from CCK-8, IC50 values, qPCR, and western blot were analyzed using GraphPad Prism 5.0 software (GraphPad Prism, San Diego, CA). Student’s t-test was utilized to compare differences between two groups. One-way ANOVA was applied for comparisons among three or more groups. Statistical signifcance was accepted for p < 0.05.

## Results

### Hy effect on HeLa and C-33A cell proliferation

**Fig. 1:**
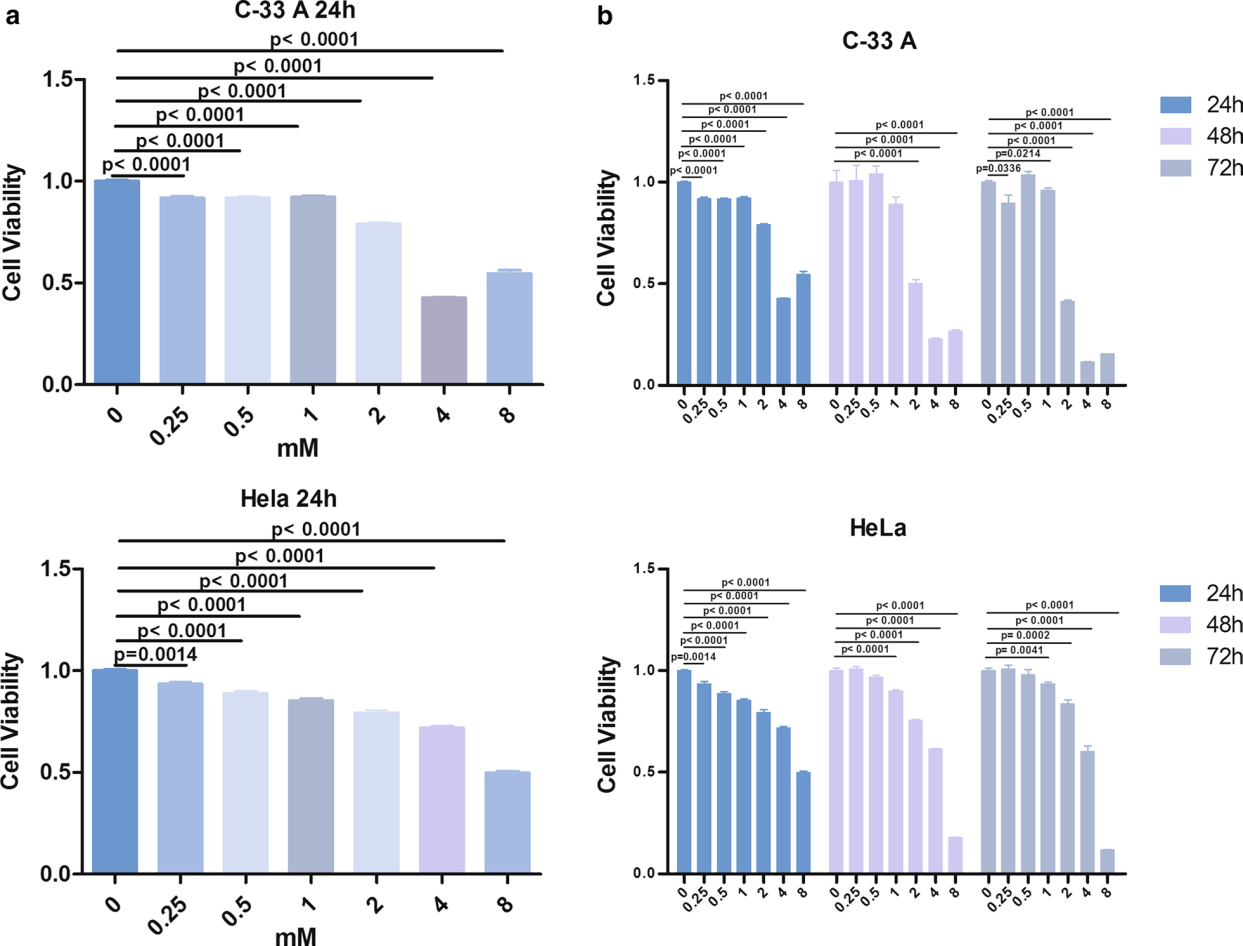
**a** Proliferation of HeLa and C-33A cells treated with a range of Hy concentrations or untreated controls at 24 h. **b** Proliferation of HeLa and C-33A cells at different time points and concentrations

After 24 h in culture, the proliferation rate of HeLa cells decreased by 6.60%, 11.37%, 14.68%, 20.65%, 28.24%, and 50.16% (P < 0.01) in the presence of 0.25, 0.5, 1, 2, 4, and 8 mM Hy, respectively, compared to that of the control group (Fig. 1a). The respective rates for C-33A cells were 8.19%, 8.33%, 7.87%, 21.09%, 57.26%, and 45.4% (P < 0.01). Furthermore, HeLa and C-33A cell viability decreased significantly with time (24, 48, and 72 h; Fig. 1b). Thus, Hy inhibited the proliferation of HeLa and C-33A cells in a dose- and time-dependent manner in vitro. The IC50 of Hy was 2 mM for C-33A cells and 4 mM for HeLa cells (Fig. 1b). Subsequent experiments included C-33A cells and 2 mM Hy.

### Sequencing data analyses

**Fig. 2:**
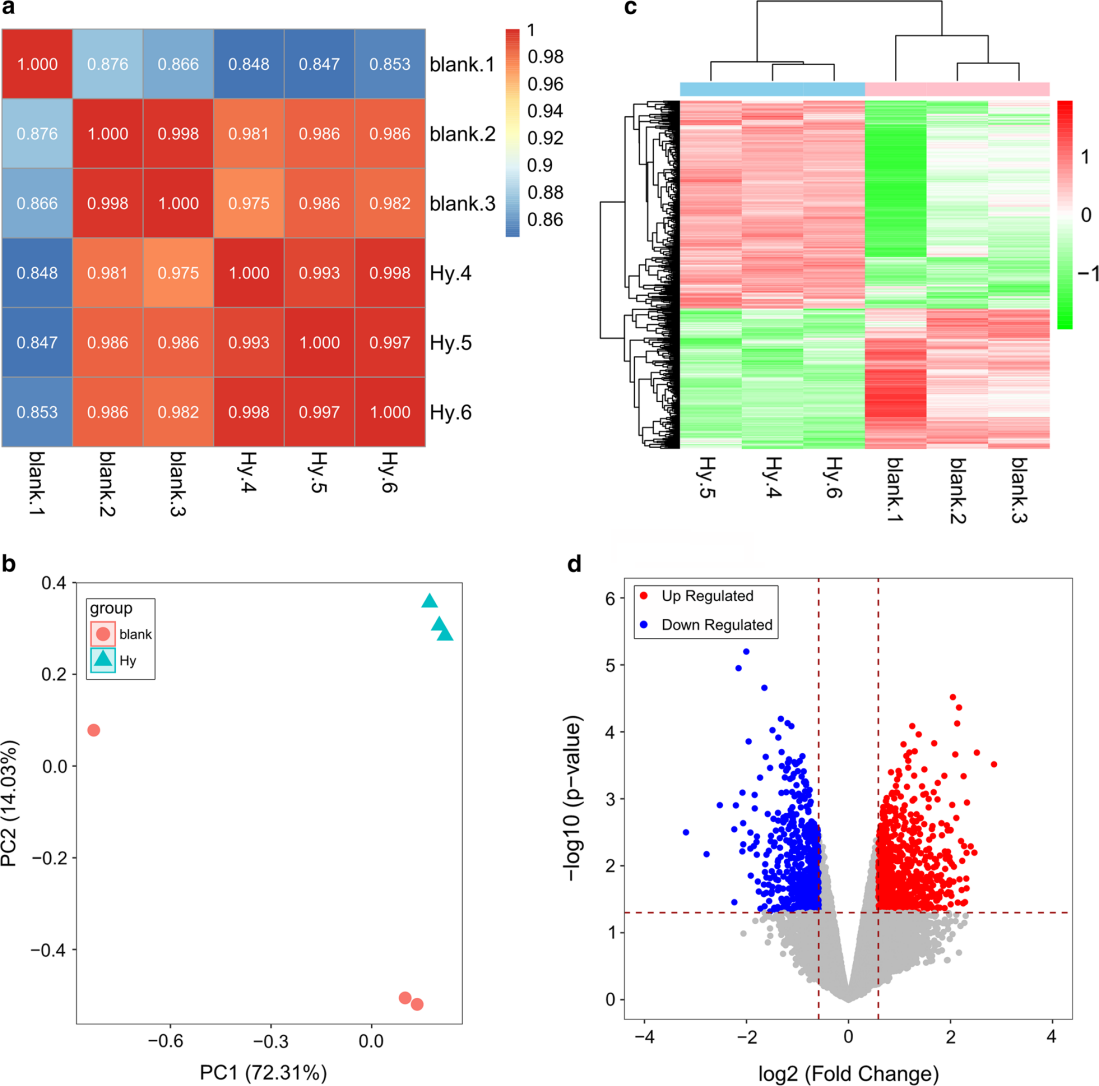
**a** Correlation heat map between pairs based on expression abundance. The darker the color, the higher the correlation; the lighter the color, the lower the correlation. **b** Principal component analysis data. **c** Cluster heat map. The top pink bar indicates the control group and the light blue bar indicates the Hy treatment group. A change in color from green to red notes that the expression level of the gene is relatively high. **d** Differential gene volcano map. Blue indicates downregulated genes and red indicates upregulated genes

After data processing, 14,000 genes were finally obtained. Based on the expression levels in each provided sample, the Pearson correlation coefficient between two samples is represented by an (r) value (Fig. 2a). The closer an (r) value is to 1, the higher the expression pattern similarity between samples. The average intragroup sample similarity was 0.977, whereas the average between-group sample similarity was 0.93. These data indicated that the samples were reasonable and the experimental results were reliable.

The results from a principal component analysis are shown in Fig. 2b. The Hy group was clearly distinct from the control group, with obvious DEGs in the Hy group and control group.

### DEG analysis

Using the defined threshold, we obtained 1263 DEGs, including 754 upregulated and 509 downregulated genes. Based on a two-dimensional hierarchical clustering heat map of the 1263 DEG values (Fig. 2c), these genes clearly separated the samples in the pre-grouping (Fig. 2d).

### Functional and pathway enrichment analysis

The 1263 DEGs were used for GO biological processes (BP) functional and KEGG pathway analyses (Table 2). The GO_BP functional analysis determined that the downregulated DEGs were mainly enriched in mitochondrial translational elongation, mitochondrial translational termination, ribosomal large subunit biogenesis, and rRNA processing, and so on. Upregulated DEGs were mainly enriched in cell adhesion, cell division, mitotic cytokinesis, and homeostasis of cell types within a tissue, etc. The KEGG pathway analysis revealed that the downregulated DEGs were mainly enriched in RNA transport, p53 signaling pathway, and transcriptional misregulation in cancer. Upregulated DEGs were enriched in endocytosis and in the PPAR, p53, GnRH, and neurotrophin signaling pathways.

**Table 2:** Key GO biological processes and KEGG pathways

DOWN or up	KEGG&GO_BP	Description	Gene
Down	KEGG_PATHWAY	hsa03013:RNA transport	*RPP38*, *NXT1*, *RPP25*, *PHAX*, *ELAC1*, *EIF1*, *PYM1*, *GEMIN6*, *POP7*
		hsa04115:p53 signaling pathway	*BID*, *SIAH1*, *PMAIP1*, *IGFBP3*, *TP53AIP1*
		hsa05202:Transcriptional misregulation in cancer	*CEBPA*, *CEBPB*, *HIST1H3E*, *IGFBP3*, *MYC*, *ATF1*, *DDIT3*, *ETV4*
	GO_BP	GO:0070125 ~ mitochondrial translational elongation	*MRPS26*, *MRPS34*, *MRPL12*, *MRPS33*, *TSFM*, *MRPS12*, *MRPL36*, *MRPS6*, *MRPL58*, *MRPL44*
		GO:0070126 ~ mitochondrial translational termination	*MRPS26*, *MRPS34*, *MRPL12*, *MRPS33*, *MRPS12*, *MRPL36*, *MRPS6*, *MRPL58*, *MRPL44*
		GO:0042273 ~ ribosomal large subunit biogenesis	*WDR74*, *NOP16*, *NIP7*, *RRS1*, *YAE1D1*
		GO:0006364 ~ rRNA processing	*RPP38*, *RPP25*, *RRP1*, *EXOSC4*, *BYSL*, *EXOSC5*, *PNO1*, *NOB1*, *RPS15A*, *DIEXF*, *LTV1*, *MRTO4*, *MRM3*
		GO:0042102 ~ positive regulation of T cell proliferation	*HAVCR2*, *HES1*, *TNFSF13B*, *ZP3*, *CD274*, *IL12A*
		GO:0070059 ~ intrinsic apoptotic signaling pathway in response to endoplasmic reticulum stress	*CEBPB*, *CHAC1*, *TRIB3*, *PMAIP1*, *DDIT3*
Up	KEGG_PATHWAY	hsa03320:PPAR signaling pathway	*ACOX2*, *ACSL1*, *EHHADH*, *RXRA*, *SCD*, *FADS2*, *GK*, *SCD5*, *ACSL3*, *ACAA1*
		hsa04115:p53 signaling pathway	*CDKN1A*, *CCNB2*, *CCND2*, *RRM2*, *APAF1*, *CCNG2*, *SESN1*, *GTSE1*, *SESN3*
		hsa04144:Endocytosis	*FGFR2*, *PRKCZ*, *LDLR*, *RAB5B*, *CYTH2*, *EEA1*, *PSD2*, *CLTC*, *GBF1*, *TFRC*, *CXCR4*, *VPS35*, *WIPF1*, *BIN1*, *CLTCL1*, *HSPA8*, *SH3GL2*, *IQSEC2*, *F2R*
		hsa04912:GnRH signaling pathway	*MAPK14*, *ADCY5*, *MAP3K1*, *CALM3*, *PRKACA*, *PRKACB*, *CACNA1F*, *CACNA1D*, *PRKCB*
		hsa04722:Neurotrophin signaling pathway	*MAGED1*, *RPS6KA2*, *MAPK14*, *BCL2*, *MAP3K1*, *CALM3*, *SORT1*, *NGFR*, *KIDINS220*, *PIK3R3*
	GO_BP	GO:0007155 ~ cell adhesion	*NRP2*, *MTSS1*, *ACHE*, *PCDHA2*, *L1CAM*, *PCDHGC3*, *PCDHAC1*, *COMP*, *COL12A1*, *CD24*, *LOXL2*, *BOC*, *APBA1*, *TYRO3*, *FLOT2*, *PODXL*, *MFGE8*, *TINAGL1*, *CTNNA1*, *COL16A1*, *MCAM*, *COL5A1*, *NCAM1*, *JUP*, *DSG2*, *CNTN1*, *SUSD5*, *ADAM12*, *NCAN*, *CD226*, *NTM*
		GO:0051301 ~ cell division	*SEPT4*, *SEPT1*, *GNAI2*, *NEK2*, *CLTC*, *CCNG2*, *CD2AP*, *SPC25*, *CDCA8*, *NCAPH*, *NCAPG*, *NCAPG2*, *CENPC*, *BUB1*, *CABLES2*, *TUBA1A*, *TUBA1B*, *CCNA2*, *CDK14*, *KIF11*, *TPX2*, *CDC20*, *RB1*, *KNSTRN*, *CDC25C*, *CDC25B*, *CCNB2*, *CCND2*, *CDCA7L*, *MAPRE2*
		GO:0000281 ~ mitotic cytokinesis	*KIF4A*, *NUSAP1*, *ANLN*, *CEP55*, *RACGAP1*, *KIF20A*
		GO:0048873 ~ homeostasis of number of cells within a tissue	*CORO1A*, *BCL2*, *ILDR2*, *F2R*, *ADD1*, *FLT3LG*
		GO:0007265 ~ Ras protein signal transduction	*ZNF304*, *CDKN1A*, *DOK3*, *MAPK14*, *IQGAP3*, *RB1*, *CCNA2*, *DHCR24*
		GO:0032012 ~ regulation of ARF protein signal transduction	*GBF1*, *CYTH2*, *PSD2*, *IQSEC2*
		GO:0045746 ~ negative regulation of Notch signaling pathway	*PEAR1*, *BEND6*, *GDPD5*, *DLK2*, *BMP7*

### A PPI network and module mining of DEGs

**Fig. 3:**
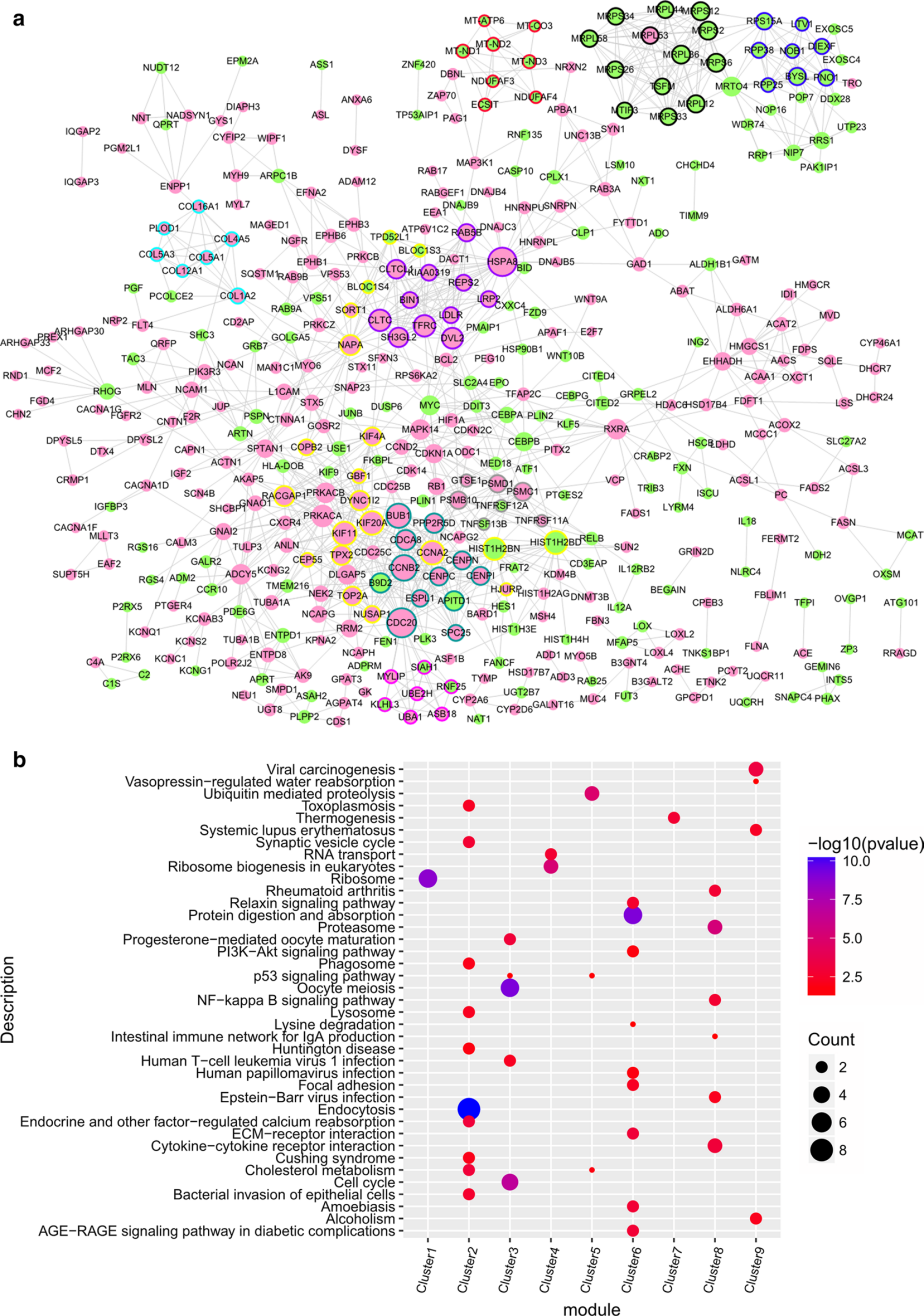
**a** A protein interaction diagram of the up- and downregulated genes. The red node indicates up-regulation and the green node indicates down-regulation. The node size illustrates the size of the interaction. The different colors of the outer edge of the node represent the score > 5 network module obtained by MCODE. **b** Sub-network module pathway analysis results. Color change from red to blue indicates a significant decrease in the P-value. The bubble size illustrates the proportion of the number of enriched genes in the corresponding module

A PPI network was obtained for a total of 435 nodes and 1130 relationship pairs (Fig. 3a). A Cytoscape software CytoNCA plug-in was used to analyze the topological properties of the DEGs in the network. The top 20 degree centrality (DC), betweenness centrality (BC), and closeness centrality (CC) scores included CCNA2, CLTC, DVL2, HIST1H2BD, HIST1H2BN, HSPA8, PRKACA, and TFRC (Table 3), which were key node proteins in the PPI network.

**Table 3:** Top 20 PPI network topology property scores

Gene	Degree	Gene	Betweenness	Gene	Closeness
CDC20	30	HSPA8	18026.846	CLTC	0.009112669
HSPA8	29	RXRA	15975.44	MYC	0.009111905
CCNB2	25	MAPK14	13874.259	TFRC	0.009106359
BUB1	23	MYC	12210.541	MAPK14	0.009104831
KIF11	22	CLTC	10207.014	CCNA2	0.009104831
KIF20A	22	TFRC	9083.066	RB1	0.00910025
CCNA2	22	DVL2	8572.19	SH3GL2	0.009099677
HIST1H2BD	21	CCNA2	8557.233	CEBPB	0.009098914
HIST1H2BN	20	MAP3K1	7784.2114	HIST1H2BD	0.009098342
CLTC	19	COL1A2	7332.0977	PRKACA	0.009098151
TFRC	19	PRKACA	7097.15	HIST1H2BN	0.009098151
DVL2	18	NAPA	6623.3286	HSPA8	0.009097007
PRKACA	18	HIST1H2BD	6509.3853	DVL2	0.009096816
CDCA8	17	CDC20	6327.5425	PRKACB	0.009096434
NAPA	17	NCAM1	6277.285	CEBPA	0.009096053
RACGAP1	17	PRKACB	6150.9224	SLC2A4	0.009095863
PPP2R5D	16	COL4A5	6065.6426	RXRA	0.009095673
DYNC1I2	16	L1CAM	5992.727	PSMC1	0.00909491
B9D2	16	HMGCS1	5969.1714	KIF4A	0.009094719
SH3GL2	16	HIST1H2BN	5857.3853	PSMB10	0.009094338

In this network, a total of 32 functional sub-modules were identified, including nine with a score > 5 (Fig. 3a). KEGG_pathway enrichment was performed on the nine modules (Fig. 3b), which were enriched mainly in: module 1—ribosomes; module 2—endocytosis; module 3—oocyte meiosis; module 4—ribosome biogenesis in eukaryotes; module 5—ubiquitin-mediated proteolysis; module 6—protein digestion and absorption; module 7—thermogenesis; module 8—proteasome; and module 9—viral carcinogenesis.

### TF prediction

**Fig. 4:**
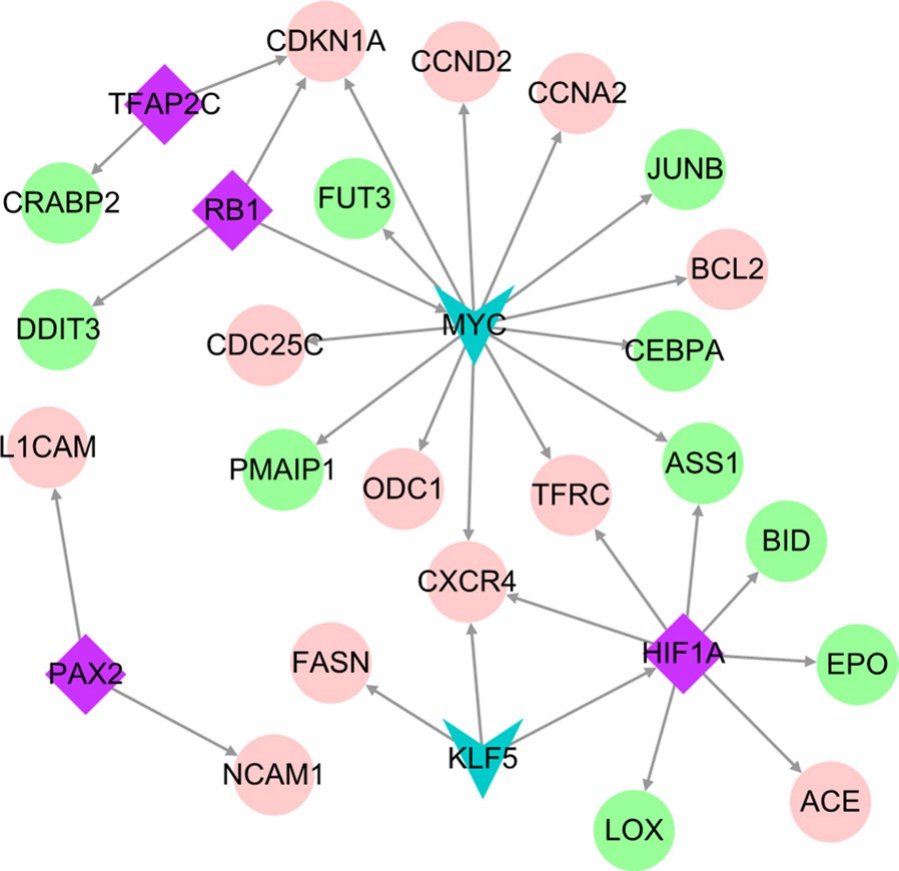
TF-target regulation network analysis map. The pink circle and green circle indicate an upregulated gene and a downregulated gene, respectively; the purple diamond and blue inverted triangle indicate an upregulated transcription factor and a down-regulated transcription factor (number of target genes ≥ 2), respectively; and the gray arrow denotes a transcription factor regulatory target gene

For TF prediction, a total of 67 TFs were obtained. With reference to the DEGs, six differentially regulated TFs were identified, which included four upregulated and two downregulated TFs. They were combined with 24 upregulated genes. In the TF-target network, *CDKN1A*, *ASS1*, *CXCR4*, and *TFRC* were coincidentally regulated by two or three TFs, which may be important for the transcriptional regulation (Fig. 4). Therefore, *CDKN1A*, *ASS1*, *CXCR4*, and *TFRC* were identified as key genes.

### Survival analysis of key genes

**Fig. 5:**
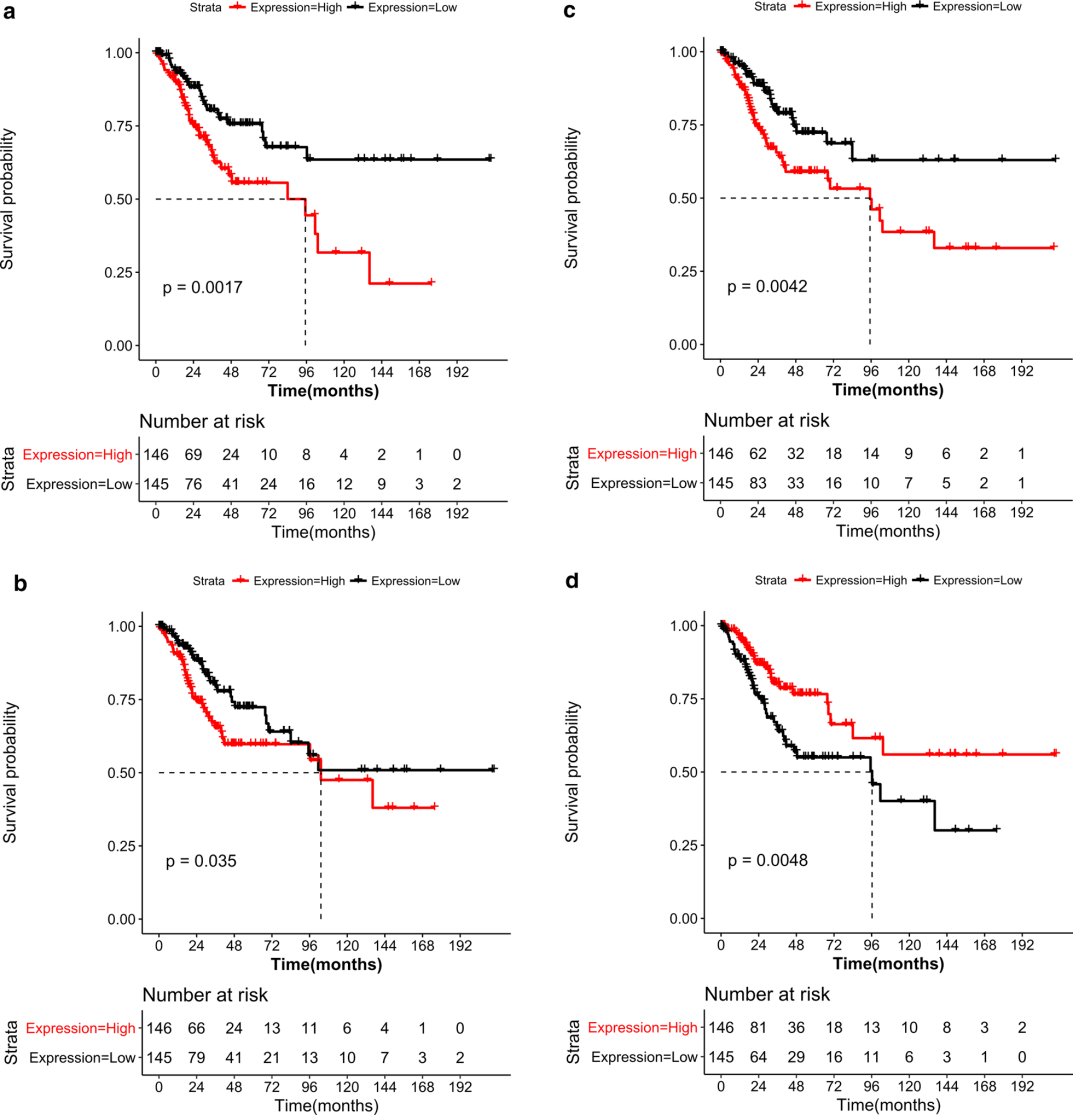
Survival curves for the key genes *CLTC* (**a**), *HSPA8* (**b**), *MYC* (**c**), and *PRKACA* (**d**); red for the high-risk group and black for the low-risk group

Based on the gene expression values and the TCGA cervical cancer clinical information, four genes were significantly associated with disease prognosis (P < 0.05). Among these, *MYC* was downregulated, whereas *HSPA8*, *CLTC,* and *PRKACA* were upregulated (Fig. 5). A survival curve analysis revealed that increased *HSPA8*, *CLTC,* and *MYC* expression and decreased *PRKACA* expression were associated with a worse prognosis.

### RT-qPCR and western blot analysis of key genes

**Fig. 6:**
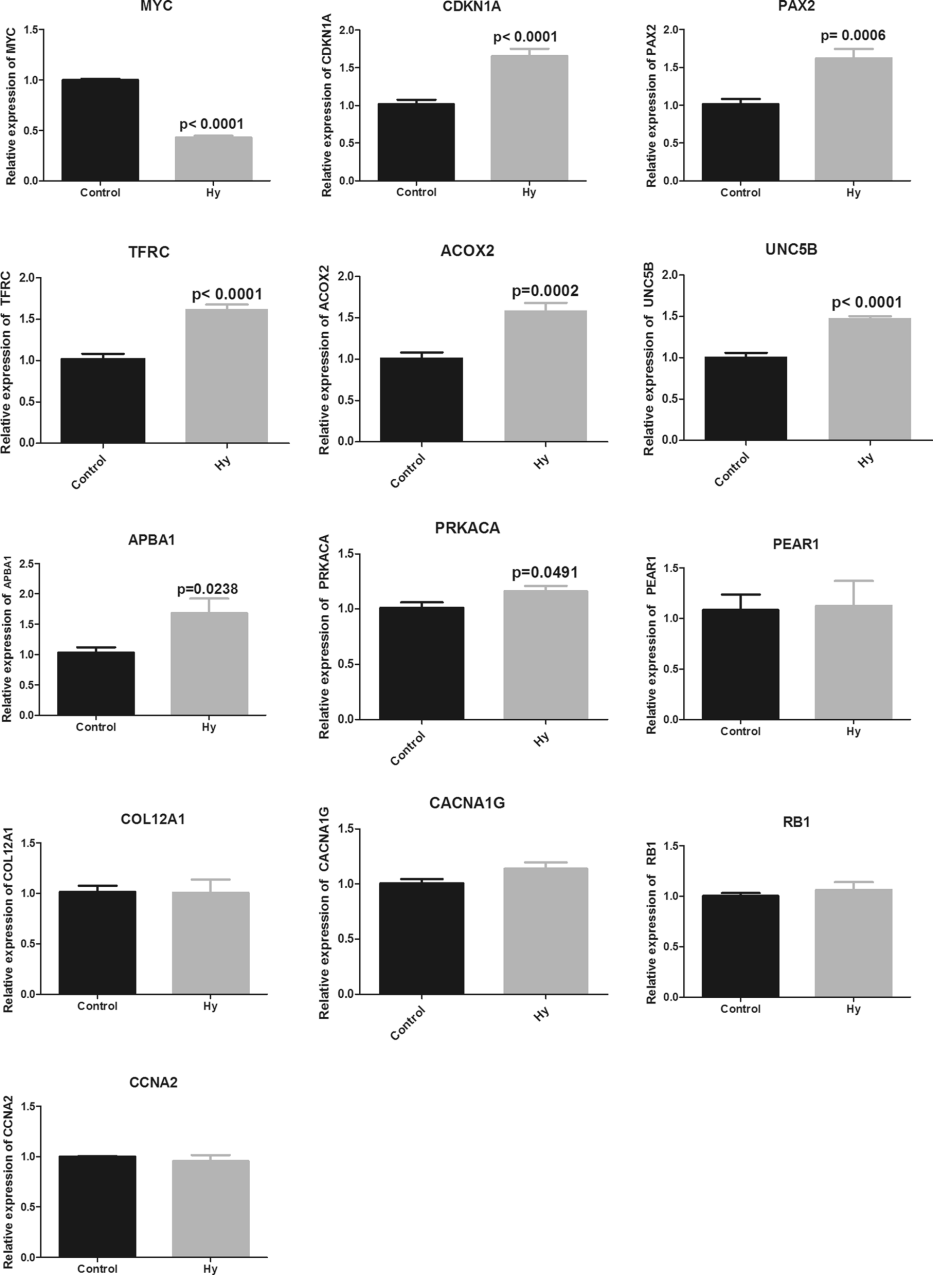
*MYC*, *CNKN1A*, *PAX2*, *TFRC*, *ACOX2*, *UNC5B*, *APBA1*, *PRKACA*, *PEAR1*, *COL12A1*, *CACNA1G*, *RB1*, and *CCNA2* mRNA expression in C-33A cells

*MYC* gene expression in Hy-treated cells was significantly downregulated (P < 0.01), whereas *CDKN1A*, *PAX2*, *TFRC*, *ACOX2*, and *UNC5B* gene expression was significantly upregulated (P < 0.01) in comparison with the blank control group measured by RT-qPCR. Moreover, *APBA1* and *PRKACA* gene levels were increased (P < 0.05). However, *PEAR1*, *CCNA2*, *COL12A1*, *PEAR1*, and *CACNA1G* did not exhibit significant changes (Fig. 6).

**Fig. 7:**
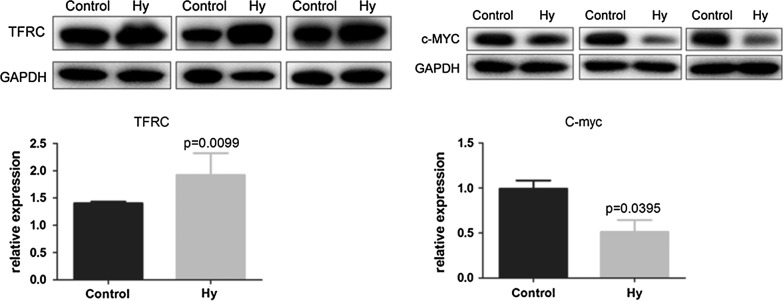
Expression of C-MYC and TFRC proteins in C-33A cells. GAPDH is the reference protein

Western blot analysis revealed that Hy treatment significantly downregulated C-MYC protein levels (P < 0.01) and significantly increased TFRC protein levels in C-33A cells compared to those in the control group (P < 0.01) (Fig. 7).

## Discussion

Hy significantly inhibited C-33A and HeLa human cervical cancer cell proliferation in a dose- and time-dependent manner. This finding is consistent with the previously described Hy-induced inhibition of human non-small cell carcinoma [26]. The mechanism of cell proliferation inhibition was further investigated in C-33 A cells. A total of 1263 DEGs were obtained by RNA-Seq and screening, indicating a significant effect of Hy on C-33A cell transcription. The identified DEGs were examined by GO_BP functional and KEGG pathway analyses. The upregulated genes were mainly enriched in cell adhesion [27], cell division and proliferation [28], peroxisome proliferator-activated receptor [29], p53 [30], and gonadotropin-releasing hormone signaling pathways [31, 32]. These signaling pathways are closely related to tumor cell migration or invasion. The downregulated genes were mainly involved in apoptosis [33], mitochondrial translation [34], ribosome-related biological processes [35], the p53 signaling pathway [36], and transcriptional dysregulation pathway [37]. It should be noted that these genes are closely related to the occurrence and development of tumors. PPI network analysis identified *CCNA2*, *CLTC*, *DVL2*, *HIST1H2BD*, *HIST1H2BN*, *HSPA8*, *PRKACA,* and *TFRC* as candidate genes. Module analysis, transcription factor prediction, and TF-target regulatory network construction selected *CDKN1A*, *ASS1*, *CXCR4*, *HIF1A*, *KLF5*, *MYC*, *PAX2*, *RB1*, and *TFAP2C*. Finally, key genes were identified by logFC, degree ranking, and survival analysis results. We selected MYC proto-oncogene (*MYC*), cyclin dependent kinase inhibitor 1A (*CDKN1A*), paired box 2 (*PAX2*), transferrin receptor (*TFRC*), acyl-CoA oxidase 2 (*ACOX2*), unc-5 netrin receptor B (*UNC5B*), amyloid beta precursor protein binding family A member 1 (*APBA1*), protein kinase cAMP-activated catalytic subunit alpha (*PRKACA*), platelet endothelial aggregation receptor 1 *(PEAR1*), collagen type XII alpha 1 chain (*COL12A1*), CACNA1G antisense RNA 1 (*CACNA1G*), RB transcriptional corepressor 1 (*RB1*), and cyclin A2 (*CCNA2*) for RT-qPCR verification in the Hy and control groups. *MYC*, *CNKN1A*, *PAX2*, *TFRC*, *ACOX2*, *UNC5B*, *APBA*1, and *PRKACA* exhibited significant differences and were consistent with previous gene screening analysis results. Then, we conducted in-depth research on *MYC* and *TFRC.* Western blot confirmed that the *MYC* gene was significantly downregulated and the *TFRC* gene was significantly upregulated.

The *MYC* gene encompasses a group of oncogenes including *C*-*MYC*, *N*-*MYC*, and *L*-*MYC* [38]. The *MYC* gene family and its products promote cervical cancer cell proliferation, immortalization, dedifferentiation, and transformation [39, 40]; furthermore, they can be used as a potential diagnostic indicator for cervical cancer. Increased *C*-*MYC* positive rate and corresponding histology findings have been correlated with cancer diagnosis [41]. Presently, RNA-Seq and DEG screening identified *MYC* as a downregulated gene. Furthermore, MYC has been critically positioned in the constructed TF-target network; it has been implicated in the regulation of multiple genes, and is significantly associated with prognosis in survival analysis [42]. RT-qPCR and western blot results further confirmed the decreased *MYC* expression in Hy-treated C-33A cells. Thus, Hy has a significant inhibitory effect on the *MYC* gene in cervical cancer C-33A cells.

*TFRC* is the most important pathway for cellular iron absorption [43]. There is increasing evidence that *TFRC* is involved in tumorigenesis and tumor progression, and its expression is significantly dysregulated in many cancer types [44]. Furthermore, *TFRC* has been closely related to human cervical cancer and is positively associated with the clinical stage and with the presence of pelvic lymph node metastases [45]. In the current study, we constructed a PPI network for DEGs and confirmed the importance of *TFRC* in the PPI. RT-qPCR and western blot analyses revealed increased *TFRC* expression after Hy treatment. The TF-target network analysis identified *TFRC* regulation by the hypoxia-inducible factor-1A (HIF-1A) signaling pathway, and elevated HIF-1A expression. Furthermore, prior studies have reported *TFRC* regulation by the HIF-1A signaling pathway. Under specific conditions, such as oxidative stress, inflammation, and hypoxia, HIF-1A expression induces binding of iron regulatory protein 1 and 2, and HIF-1A promotes *TFRC* transcription. Furthermore, HIF-1A regulates *TFRC* transcription by DNA binding, and the subsequent *TFRC* protein production promotes iron metabolism and increases oxygen exchange [44]. However, the mechanism by which Hy increases *TFRC* expression remains elusive. It may be due to high HIF-1A levels caused by hypoxia. Perhaps Hy is unlikely to inhibit the TFRC-related HIF-1A signaling pathway in C-33A cell. The mechanism, by which Hy inhibits tumor proliferation, requires further experimentation and discussion.

## Conclusions

In summary, Hy inhibits HeLa and C-33A cervical cancer cell proliferation, and regulates the transcription process in C-33A cells. These findings provide a new avenue for the clinical treatment of cervical cancer and a theoretical basis for the clinical application of Hy.

## Data Availability

Not applicable.

## Abbreviations

Hy: hyperoside
DEGs: differentially expressed genes
TF: transcription factor
GO: gene ontology
BP: biological processes
KEGG: Kyoto Encyclopedia of Gene and Genome
HPV: human papillomavirus
NF-κB: nuclear factor-kappa B
RNA-seq: RNA sequencing
IC50: half-inhibitory concentration
*MYC*: MYC proto-oncogene
*CDKN1A*: cyclin dependent kinase inhibitor 1A
*PAX2*: paired box 2
*TFRC*: transferrin receptor
*ACOX2*: acyl-CoA oxidase 2
*UNC5B*: unc-5 netrin receptor B
*APBA1*: amyloid beta precursor protein binding family A member 1
*PRKACA*: protein kinase cAMP-activated catalytic subunit alpha
*PEAR1*: platelet endothelial aggregation receptor 1
*COL12A1*: collagen type XII alpha 1 chain
*CACNA1G*: CACNA1G antisense RNA 1
*RB1*: RB transcriptional corepressor 1
*CCNA2*: cyclin A2
HIF-1A: hypoxia-inducible factor-1A
